# Musculoskeletal Dimension and Brightness Reference Values in Lumbar Magnetic Resonance Imaging—A Radio-Anatomic Investigation in 80 Healthy Adult Individuals

**DOI:** 10.3390/jcm13154496

**Published:** 2024-08-01

**Authors:** Horst Balling, Boris Michael Holzapfel, Wolfgang Böcker, Dominic Simon, Paul Reidler, Joerg Arnholdt

**Affiliations:** 1 Department of Orthopaedics and Trauma Surgery, Musculoskeletal University Center Munich (MUM), University Hospital, Ludwig-Maximilians-Universität Munich, Marchioninistr. 15, 81377 Munich, Germanyjoerg.arnholdt@med.uni-muenchen.de (J.A.); 2 Center for Spine Surgery, Neckar-Odenwald-Kliniken gGmbH Buchen, Dr.-Konrad-Adenauer-Str. 37, 74722 Buchen, Germany; 3 Department of Radiology, University Hospital, Ludwig-Maximilians-Universität Munich, Marchioninistr. 15, 81377 Munich, Germany

**Keywords:** MRI, lumbar spine, aging, paraspinal muscles, psoas muscle, dimension, brightness

## Abstract

**Background/Objectives**: Magnetic resonance imaging (MRI) is the preferred diagnostic means to visualize spinal pathologies, and offers the possibility of precise structural tissue analysis. However, knowledge about MRI-based measurements of physiological cross-sectional musculoskeletal dimensions and associated tissue-specific average structural brightness in the lumbar spine of healthy young women and men is scarce. The current study was planned to investigate characteristic intersexual differences and to provide MRI-related musculoskeletal baseline values before the onset of biological aging. **Methods**: At a single medical center, lumbar MRI scans of 40 women and 40 men aged 20–40 years who presented with moderate nonspecific low back pain were retrospectively evaluated for sex-specific differences in cross-sectional sizes of the fifth lumbar vertebrae, psoas and posterior paravertebral muscles, and respective sex- and age-dependent average brightness alterations on T2-weighted axial sections in the L5-level. **Results**: In women (mean age 33.5 years ± 5.0 (standard deviation)), the investigated musculoskeletal cross-sectional area sizes were significantly smaller (*p* < 0.001) compared to those in men (mean age 33.0 years ± 5.7). Respective average musculoskeletal brightness values were higher in women compared to those in men, and most pronounced in posterior paravertebral muscles (*p* < 0.001). By correlating brightness results to those of subcutaneous fat tissue, all intersexual differences, including those between fifth lumbar vertebrae and psoas muscles, turned out to be statistically significant. This phenomenon was least pronounced in psoas muscles. **Conclusions**: Lumbar musculoskeletal parameters showed significantly larger dimensions of investigated anatomical structures in men compared to those in women aged 20–40 years, and an earlier onset and faster progress of bone loss and muscle degradation in women.

## 1. Introduction

Magnetic resonance imaging (MRI) is the gold standard in modern spinal imaging if musculoskeletal pathologies of the spine are to be diagnosed [1,2]. It depicts the morphology of soft tissues and osseous structures, as well as fluid collections, and helps to discern bone bruise and spinal stenosis. Structural muscle tissue degradation, including a reduction in cross-sectional size or fatty degeneration, are easily detectable with this imaging modality, and play a crucial role in the identification of, for example, rotator cuff pathologies in MRI investigations of the shoulder [3]. However, even though there is evidence that a loss of spinal muscle mass correlates to osteoporosis and low back pain [4], such changes are usually not referred to in radiologists’ reports.

In order to investigate the fatty involution of soft tissues, specific fat-suppressed imaging series are mandatory, and these are not generally included in conventional lumbar MRI studies. In particular, muscle quantity, fiber architecture, and muscle fat content are parameters used to assess the extent of sarcopenia-related muscle degradation [5], but it is still unclear how physiologic threshold values can be defined to separate healthy adult women and men from those suffering from complications related to osteoporosis and sarcopenia. Measurable correlations between age-dependent structural musculoskeletal changes in MRI and osteoporosis- or sarcopenia-related tissue degradation might be reflected by a loss of cross-sectional muscle dimensions, or a change in tissue brightness. In T2-weighted MRI sequences, for example, increasing contents of intramuscular fat lead to higher intramuscular brightness values, which might be indicative of the aging process if relevant degenerative alterations have been ruled out. Moreover, an increasing loss of muscle strength at higher ages might not only be an effect of fatty degeneration of the muscle tissue, but could also result from a measurable loss of cross-sectional muscle mass with an onset at a time point that could mark the beginning of seniority initiated through a less active lifestyle [6,7]. In recent years, several authors have investigated the effect of symptomatic spinal pathology, sex, or aging on paraspinal muscle size and fatty infiltration [8,9,10]. But these measurements are rather technical (for scientific applications), and require specific software.

In a clinical setting, however, the conventional MRI brightness measuring tool from the clinical Picture Archiving and Communication System (PACS) is more practicable. Unfortunately, there are no reference data available in the literature on tissue brightness in T2-weighted MRI investigations of healthy non-geriatric adults. Therefore, this radio-anatomic MRI-based investigation was initiated to define reference values for cross-sectional muscle and vertebral body dimension measurements, and for structural tissue brightness determination in young adults. We hypothesized that T2-weighted MRI-based cross-sectional musculoskeletal dimension and brightness data from a pre-seniority collective of healthy individuals could serve as reference values for future research.

## 2. Materials and Methods

### 2.1. Formal Study Prerequisites

The study protocol was approved by the clinic’s medical ethics committee (Ref.-Nr. 23-0362), in accordance with the WMA (World Medical Association) Declaration of Helsinki from 1964, including its later amendments. Informed consent from participating individuals was not required due to anonymized data collection in this retrospective study.

The authors have work experience of 7 to over 15 years, with a main focus on the treatment of geriatric individuals.

### 2.2. Participant Recruitment

Radiologic data from individuals who presented to the emergency room of a general hospital to receive medical treatment after minor extremity or chest trauma from 1 June 2020 to 4 August 2022 were reviewed for recent or previous lumbar MRI studies. Adult subjects who fulfilled the inclusion criteria but did not meet exclusion criteria (Table 1) were collected in a database until data sets from 40 women and 40 men, aged 20–40 years, had been completed. 

### 2.3. Technical Approach of Measurements and Underlying Theoretical Considerations

**Figure 1:**
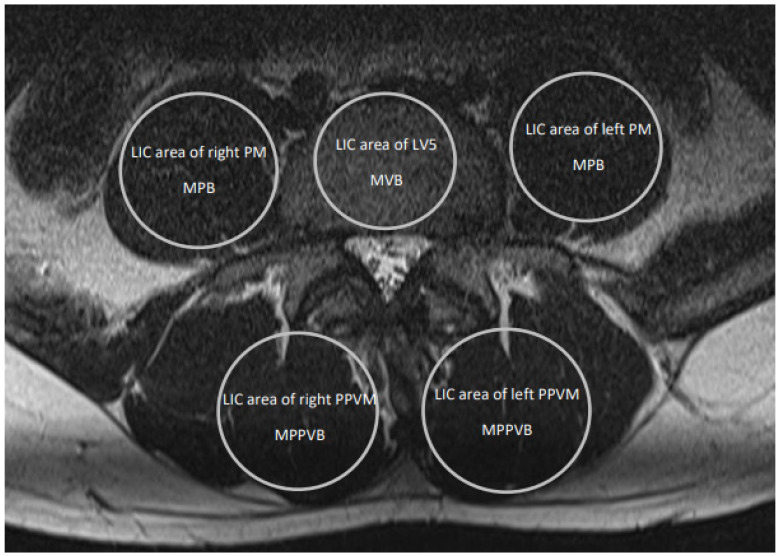
Example showing measurement schemes of LIC area size and brightness in the fifth lumbar vertebral body, with both psoas and both erector spinae muscles. Axial MRI slice through the lower lumbar spine showing vertebral and muscle structures of a 39-year-old man without spinal pathologies. LIC indicates largest inscribed circle; MRI, magnetic resonance imaging; L5, lumbar vertebra V; PM, psoas muscle; PPVM, posterior paravertebral muscle; MVB, mean vertebral (body) brightness; MPB, mean psoas (muscle) brightness; MPPVB, mean posterior paravertebral (muscle) brightness.

Eligible MRI studies had to be available in the clinic’s PACS (RVC Clinical PACS^®^, version 22.2.1 14467, Freiburg, Germany) to allow measurements on transaxial slices provided in DICOM (Digital Imaging and Communications in Medicine) format. In order to avoid methodological bias during the comparison of results from different individuals, exclusively T2-weighted image series were assessed. All measurements were conducted in a horizontal plane, cutting parallel through the upper half of the L5-level. Cross-sectional psoas muscle (PM), posterior paravertebral muscle (PPVM), and fifth lumbar vertebral body (LV5) area sizes were determined approximately with the PACS-integrated circle area and brightness measuring tool, using the geometric shape of a circle as the best fitting largest inscribed circle (LIC) in respective anatomical regions of interest (ROIs), as shown in Figure 1.

Using this technique, five sets of values from LV5, both PMs, and both PPVMs were obtained, including cross-sectional area size, mean brightness (i.e., signal intensity) values with minimum and maximum values, and standard deviations of respective ROIs.

All MRI scans were performed with a 1.5-Tesla scanner (Magnetom Avanto, Tim, 76 × 18 Q-engine, Siemens Healthineers, Erlangen, Germany). Time to echo was 82.0–84.0 ms, repetition time was 2780.0–8894.9 ms, field of view was 240 mm × 240 mm to 260 mm × 260 mm, and matrix format was 256 × 512.

### 2.4. Creation of Physiological Baseline Ranges and Normalization of Signal Intensity Values

Finally, to provide standard values for cross-sectional muscle size, vertebral body dimension, and structural tissue brightness values in women and men of a pre-seniority age group, parameter means and corresponding 95% confidence intervals (CIs) were determined to define a physiological range of values to be used for future research in this field of emerging scientific interest. In order to standardize brightness measurements, values were related to the signal intensity of subcutaneous fat tissue in T2-weighted images (normalization). Thus, results can additionally be reported as percentages of the brightness of subcutaneous fat tissue.

### 2.5. Statistics

Baseline variables were expressed as means and range of values in case of continuous data, and as percentages in case of categorical parameters. Data from bilateral muscle measurements were recorded as average values from corresponding ROIs. Student’s *t*-test was used to compare continuous variables following a normal distribution. Results were displayed as means and 95% CIs. Sex-related comparisons and all analyses were performed with SPSS 15.0.1 for Windows (SPSS Inc., Chicago, IL, USA). The significance level was set at *p* < 0.05. Cohen’s d was used to clarify the effect size of statistically significant differences. Intraclass correlation (ICC) was performed to quantify interrater reliability, and the Pearson correlation coefficient was determined to investigate interclass correlation. The first author is responsible for the sincerity and transparency of all statistical analyses, and will answer any pre- or post-publication queries.

## 3. Results

**Figure 2:**
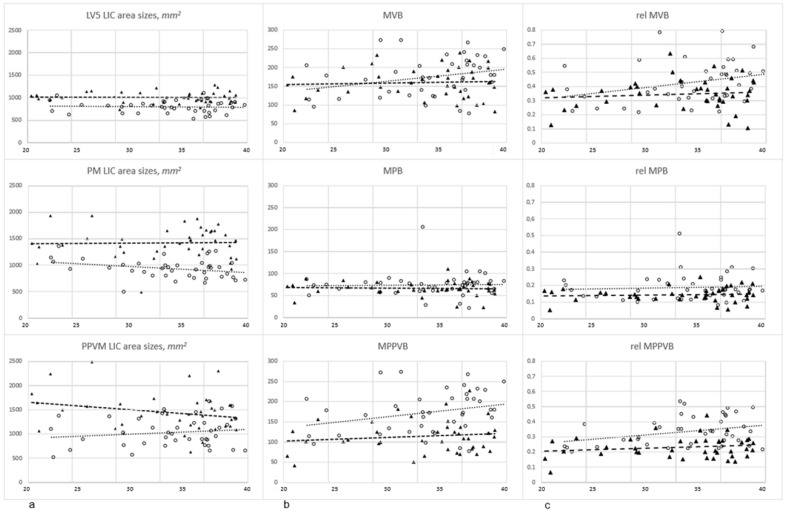
(**a**–**c**) Results from MRI-based LIC area (**a**) and mean brightness measurements (**b**) in LV5s, PMs, and PPVMs in 40 women (circles) and 40 men (triangles) ordered by increasing age (x-axis: age in years). Black dashed lines in graphs are trend lines of males’ data points, and grey dotted lines are trend lines of females’ data points. LIC areas were significantly larger in men, and PPVMs were significantly brighter in women. As soon as brightness measurements were correlated to subcutaneous fat tissues (**c**), significantly higher values resulted for all investigated structures in women. MRI indicates magnetic resonance imaging; LIC, largest inscribed circle; L5, lumbar vertebra V; PM, psoas muscle; PPVM, posterior paravertebral muscle; MVB, mean vertebral (body) brightness; MPB, mean psoas (muscle) brightness; MPPVB, mean posterior paravertebral (muscle) brightness; rel, related to the brightness of subcutaneous fat tissue.

During the recruitment period, 40 women with an average age of 33.5 (22–40) years (95% CI [31.9, 35.1]) and 40 men with a mean age of 33.0 (20–39) years (95% CI [31.2, 34.8]) were included in this investigation. Significant differences in age distribution were not detected (*p* ≈ 0.68). Women aged 20–30 years constituted 20.0%, and those aged 31–40 years made up 80.0% of female participants. In addition, 27.5% of male participants were between 20 and 30 years old, and 72.5% were between 31 and 40 years of age. No structural pathologies were found in investigated MRIs with the potential to substantially influence measurements, such as tumors, inflammatory processes, arthritis, scoliotic or kyphotic deformation, or findings of relevant muscle degradation. Musculoskeletal cross-sectional area sizes were significantly smaller in women compared to those in men (*p* < 0.001, Table 2, Figure 2a). 

**Figure 3:**
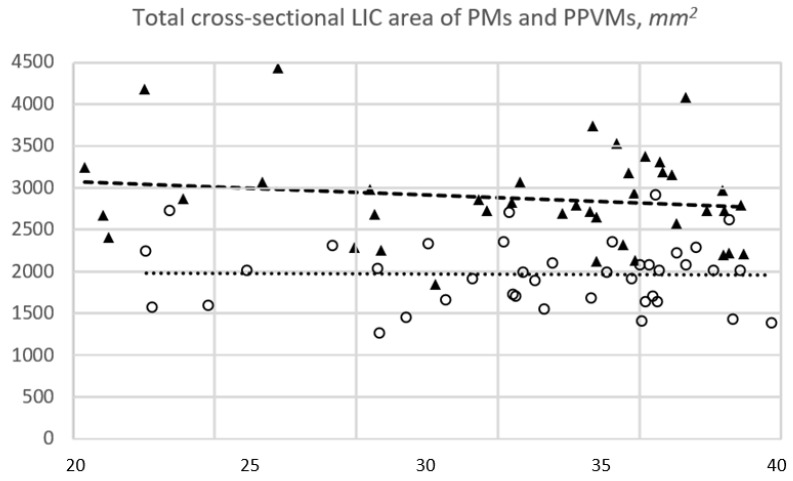
MRI-based cross-sectional muscle area measurements consisting of LIC areas of PMs and PPVMs at a horizontal plane cut parallel through the upper half of the L5-level in 40 women (circles) and 40 men (triangles) ordered by increasing age. The black dashed line is the trend line of males’ data points, and the grey dotted line is the trend line of females’ data points. Total cross-sectional muscle mass seems to be constant in women, but decreases in men with growing age (x-axis: age in years; y-axis: mm^2^). MRI indicates magnetic resonance imaging; LIC, largest inscribed circle; PM, psoas muscle; PPVM, posterior paravertebral muscle; L5, lumbar vertebra V; mm^2^, square millimeter.

These sex-related differences in LIC area sizes remained at a constant level for LV5s (women’s mean 801.4 mm^2^, 95% CI [759.6, 843.1] versus men’s mean 1004.4 mm^2^, 95% CI [967.0, 1041.7]). In women, LIC area sizes decreased with growing age for PMs (mean 932.0 mm^2^, 95% CI [874.8, 989.1]), but similarly increased for PPVMs (mean 1035.9 mm^2^, 95% CI [941.9, 1129.9]), which resulted in constant levels of total cross-sectional muscle area sizes across the investigated age range (Figure 3). 

In men, cross-sectional LIC area sizes were constant for PMs (mean 1420.5 mm^2^, 95% CI [1329.7, 1511.3]), but total cross-sectional muscle area sizes decreased in higher age groups with the amount of loss of respective PPVM-related LIC area sizes (mean 1444.5 mm^2^, 95% CI [1322.6, 1566.4], Figure 2a). 

Average musculoskeletal brightness values were higher in women at all ROIs (Figure 2b). However, average sex-related brightness differences for LV5s (*p* ≈ 0.17) and PMs (*p* ≈ 0.15) did not reach statistical significance. The only statistically significant intersexual brightness difference was seen in PPVMs (women’s mean 141.9, 95% CI [128.6, 155.1] versus men’s mean 114.1, 95% CI [101.8, 126.4], *p* < 0.001), which was accompanied by the above-mentioned age-related PPVM cross-sectional size increase in women, and the PPVM cross-sectional size decrease in men. In women, the age-related mean brightness increase was most pronounced for LV5s, less pronounced for PPVMs, and constant for PMs. In men, these brightness measurements were almost constant in investigated age groups, except for a slight age-related brightness increase for PPVMs (Figure 2b). Investigations on Cohen’s d showed medium effect sizes for statistically significant differences in average brightness values (0.6–0.7), and large effect sizes for statistically significant differences in average musculoskeletal dimensions and normalized posterior paravertebral muscle brightness values (1.2–2.0) between women and men (Table 2). 

The ICC was 0.84–0.92 for side-specific cross-sectional muscle area measurements in women and 0.89–0.93 in men. The ICC was 0.79–0.84 for side-specific muscle brightness measurements in women and 0.84–0.85 in men. The interclass correlation of measurements was positive (Pearson correlation coefficient 0.28–0.38).

**Figure 4:**
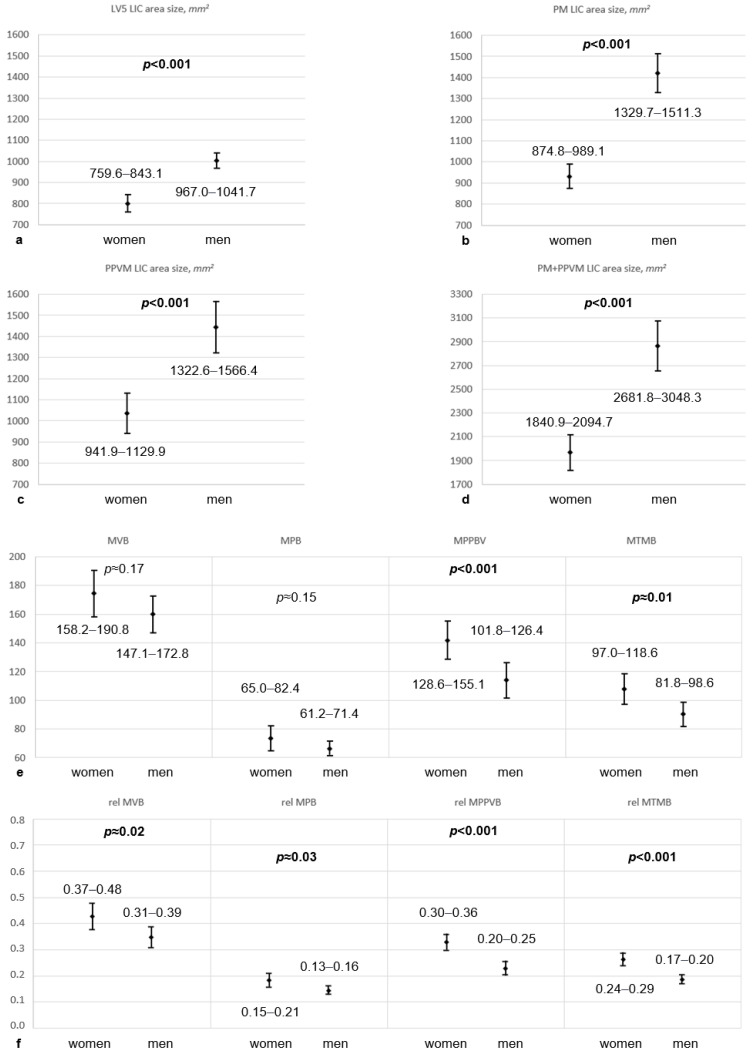
(**a**–**f**) Graphical depiction of means and 95% CIs of cross-sectional vertebral body (**a**) and muscle dimensions (**b**–**d**) at the upper half of the L5-level, and of absolute (**e**) and relative brightness (**f**) in corresponding locations, i.e., LV5, PM, PPVM, and total perivertebral muscle mass in 40 women and 40 men aged 20–40 years. L5/LV5 indicates lumbar vertebra V; 95% CI, 95% confidence interval; LIC, largest inscribed circle; mm^2^, square millimeter; PM, psoas muscle; PPVM, posterior paravertebral muscle; MVB, mean vertebral (body) brightness; MPB, mean psoas (muscle) brightness; MPPVB, mean posterior paravertebral (muscle) brightness; MTMB, mean total muscle brightness; rel, related to the brightness of subcutaneous fat tissue.

Physiological ranges of cross-sectional muscle and vertebral body dimensions in the upper half of the L5-level and of corresponding brightness values derived from presented results are displayed in Figure 4 (95% CIs) and in Table 2. Brightness values were additionally correlated to the brightness of subcutaneous fat tissue to obtain normalized values for investigated anatomical structures. After this normalization, not only were PPVM brightness values significantly higher in women than in men, but also those of LV5s and PMs (Table 2, Figure 2c and Figure 4f).

## 4. Discussion

This MRI-based investigation used quantitative methods to approximately assess the dimensions and quality of paravertebral muscles and LV5s with the circle area and brightness measuring tool from the clinical PACS software package, version 22.2.1. Other authors have performed similar quantitative analyses with computed tomography (CT) [11,12,13]. However, it is unknown how CT-specific Hounsfield unit-related density values correlate to MRI-based brightness measurements, as both radiologic modalities adhere to completely different principles of imaging. In this study, we performed tissue dimension and brightness measurements based on T2-weighted MRI sequences, which were of equivalent reliability and validity compared to measurements based on T1-weighted sequences, as has previously been shown by Cooley et al. [9].

For comparing cross-sectional muscle and vertebral body sizes, an intra-structural circle was chosen as the geometric form to obtain approximate values for determining dimensions of depicted anatomical structures. The measurements performed here naturally rather underestimated true anatomical dimensions. A more accurate method to estimate the true size of a two-dimensionally represented irregularly shaped structure would have been to calculate the arithmetic mean of the smallest outer and largest inner circle areas [14]. However, since anatomical structures are usually shown in axial MRI slices in an oblique instead of an orthogonal cross-section, the depiction on images is rather an overestimated representation of true dimensions. Therefore, more precise area measurements of depicted shapes would have led to incorrect overestimations of real anatomical dimensions. In addition, since the same underestimating measuring method was used throughout the data collection, comparative statements were possible. Moreover, with these considerations in mind, the presented measuring technique should be accurate enough to provide values close to real anatomical dimensions of musculoskeletal structures.

The current MRI-based study on cross-sectional musculoskeletal parameters was intended to reliably identify physiological baseline values in healthy adults without a history of spinal disease. Intersexual comparisons showed significant differences according to average cross-sectional sizes between women and men. Sex-specific differences were also found in CT-based investigations of other authors [15]. These differences in anatomical dimensions between women and men are usually at least partly due to the fact that women and men have a slightly different growth-onset during maturation, with different dynamics, and an earlier cessation of length-growth in women [16]. An important finding in this investigation was that in the investigated age group, constant cross-sectional sizes could be detected for PM in men, and for the entire paravertebral muscle group (PMs and PPVMs) in women. Specifically, for the PPVM size of healthy volunteers aged 20–62 years, Crawford et al. found no age dependence, but they investigated muscle volume instead of cross-sectional size [17]. Shahidi et al. also found no age dependence in paraspinal muscle size using MRI-based cross-sectional area measurements in their study collective of 199 individuals aged 18–80 years [10]. 

Average intersexual brightness differences were most pronounced in PPVMs (*p* < 0.001), but did not reach statistical significance in LV5s (*p* ≈ 0.17) or PMs (*p* ≈ 0.15). The smallest constant brightness difference was detected for the PM, which is obviously similarly strained in middle-aged women and men without showing signs of relevant age-related degeneration. The erector spinae muscle, however, seems to take a different development, reflected by an increase in brightness values in women and a decrease in cross-sectional area size in men during the third and fourth decade of life. Increasing intramuscular brightness values in T2-weighted MRI series are generally interpreted as signs of degenerative alterations to muscle tissue, which occur during aging and correlate with higher fat tissue content as an effect of irreversible muscle tissue transformation [18]. Increasing vertebral body brightness similarly mirrors the loss of vertebral bone mass, or progressive fatty bone marrow degeneration. This could mean that compared to men, women are subjected to an earlier onset of bone loss, starting as soon as their fourth decade, and that in either sex, muscular degeneration starts earlier in PPVMs than in PMs, and at a higher pace in women (Figure 2). Significantly more severe fatty infiltration in PPVMs in women compared to men was also found by other authors [4,17], who used lumbar T2- or T1-weighted MRI studies to (semi-)quantitatively analyze the degree of fat deposition in paraspinal muscle tissue. Such semi-quantitative grading adheres to a four-point visual scale, and is subjected to methodological bias and reduced inter-observer reliability [19], whereas our study uses objective data, which are reproducible apart from measurement errors of negligible extent. Several authors have differentiated between specific muscle groups of the PPVM, i.e., multifidus and erector spinae muscles, as these separate structures can degenerate at a different pace during aging [4,11,13]. In order to keep data collection simple, PPVM measurements were performed in the current study without further differentiation for separate muscle groups, which did not seem to be necessary, either, as participating individuals were aged 20–40 years without a history of spinal disease and without considerable fatty involution of the perispinal muscle groups. Nevertheless, tissue brightness values tended to be higher in women than in men. Standardized through the correlation to individual fat tissue brightness levels, muscle tissue was measured to be 1.9–12.4 times darker than fat tissue in women, and 2.3–19.5 times darker than fat tissue in men. The investigated vertebral bone was shown to be 1.3–4.7 times darker in women and 1.6–9.2 times darker in men compared to subcutaneous fat tissue in these individuals. Based on the underlying collective of healthy subjects, these interindividual brightness fluctuations were interpreted as the physiological range of varying brightness. Unfortunately, to date there have been no comparative or reference data available in the literature to discuss findings in this work.

An assumed causal pathway from spinal pathology to muscle degeneration is still under debate [20]. Some results support the hypothesis that associations exist between lumbar degenerative pathologies and lower proportions of pure multifidus muscle, indicating a potential dose–response relationship between the number of spinal pathologies and lumbar multifidus muscle morphology [21]. However, questions like these cannot be answered with this study’s collective of healthy individuals aged 20–40 years, since this investigation was intended to provide standard values derived from healthy young subjects without degenerative spinal alterations.

This study has several limitations. Individuals were not matched sex-wise as to body height or weight. All participants were Europeans. Therefore, results from this study cannot be unconditionally transferred to collectives of other ethnicities with substantially different physical constitutions. The study is lacking a control group, as it was planned only to create a reference data set of physiological baseline values to be used as a control group itself for future comparisons with, e.g., senior collectives. The study was conducted as a cross-sectional investigation rather than a longitudinal one. Therefore, findings cannot be interpreted as being predictive for the individual development of muscle degeneration, but they indicate a higher prevalence of initial loss of functional muscle tissue in higher ages, which obviously starts in the fourth decade.

The presented confines of physiological values for cross-sectional muscle and vertebral body size in the upper half of the L5-level, and for corresponding tissue brightness values in T2-weighted MRI scans of the lumbar spine, still require further validation before implementation into clinical care is possible. Nevertheless, results are suitable for future research as standard values for healthy adult women and men belonging to a pre-seniority age group.

## Figures and Tables

**Table 1:** Inclusion and exclusion criteria of the study.

Inclusion criteria	healthy individuals aged 20–40 years at the time of evaluated lumbar MRI studies AND MRI performed in supine position of the individual AND availability of lumbar MRI with axial T2 sequence showing inferior lumbar levels, both psoas muscles, and both erector spinae muscles
Exclusion criteria	individuals with a documented history of body growth disturbance, bone mineral density pathologies, or cachexia OR structural spinal pathology, such as scoliosis, fracture, disc degeneration, spinal stenosis, spondyloarthritis, etc., OR condition after spinal surgery OR evidence of the existence of tumorous lesions of the fifth lumbar vertebra or paraspinal muscles OR MRI performed in prone or lateral position of the individual

To include an individual in the study, all inclusion criteria had to be fulfilled. For the exclusion of a subject, the presence of a single exclusion criterion sufficed. MRI indicates magnetic resonance imaging.

**Table 2:** Summary of results. Differences between groups were significant for LIC area sizes, MPPVB, and normalized mean tissue brightness values. Normalized mean tissue brightness values were obtained by correlating actual brightness values to those of depicted subcutaneous fat tissue. Significance was reached at *p* < 0.05 (Student’s *t*-test), significant values are in bold.

Result	Women (*n* = 40)		Men (*n* = 40)		*p*	Effect Size
	Mean (Range)	95% CI	Mean (Range)	95% CI		Cohen’s d
LV5 LIC area size, mm^2^	801.4 (534.7–1103.6)	[759.6, 843.1]	1004.4 (737.2–1279.9)	[967.0, 1041.7]	**<0.001**	1.6
PM LIC area size, mm^2^	932.0 (489.6–1358.6)	[874.8, 989.1]	1420.5 (485.3–1939.6)	[1329.7, 1511.3]	**<0.001**	2.0
PPVM LIC area size, mm^2^	1035.9 (511.1–1670.0)	[941.9, 1129.9]	1444.5 (628.0–2489.1)	[1322.6, 1566.4]	**<0.001**	1.2
PM + PPVM LIC area size, mm^2^	1967.8 (1257.9–2926.7)	[1840.9, 2094.7]	2865.0 (1848.9–4428.4)	[2681.8, 3048.3]	**<0.001**	1.8
MVB	174.5 (77.3–272.9)	[158.2, 190.8]	160.0 (82.8–240.4)	[147.1, 172.8]	0.17	-
Normalized MVB	0.43 (0.21–0.79)	[0.37, 0.48]	0.35 (0.11–0.63)	[0.31, 0.39]	**0.02**	0.6
MPB	73.7 (20.6–205.6)	[65.0, 82.4]	66.3 (23.2–110.8)	[61.2, 71.4]	0.15	-
Normalized MPB	0.18 (0.08–0.51)	[0.15, 0.21]	0.14 (0.05–0.25)	[0.13, 0.16]	**0.03**	0.6
MPPVB	141.9 (78.2–222.6)	[128.6, 155.1]	114.1 (41.5–227.0)	[101.8, 126.4]	**<0.001**	0.7
Normalized MPPVB	0.33 (0.20–0.53)	[0.30, 0.36]	0.23 (0.06–0.44)	[0.20, 0.25]	**<0.001**	1.2
MTMB	107.8 (20.6–222.6)	[97.0, 118.6]	90.2 (23.2–227.0)	[81.8, 98.6]	**0.01**	0.7
Normalized MTMB	0.26 (0.08–0.53)	[0.24, 0.29]	0.19 (0.05–0.44)	[0.17, 0.20]	**<0.001**	0.7

CI indicates confidence interval; LV5, lumbar vertebra V; LIC, largest inscribed circle; mm^2^, square millimeter; PM, psoas muscle; PPVM, posterior paravertebral muscle; MVB, mean vertebral (body) brightness; MPB, mean psoas (muscle) brightness; MPPVB, mean posterior paravertebral (muscle) brightness; MTMB, mean total muscle brightness.

## Data Availability

The data presented in this study are available within the article and its Supplementary Materials.

## Supplementary Materials

The following supporting information can be downloaded at: https://www.mdpi.com/article/10.3390/jcm13154496/s1, Supplemental PowerPoint Slides.pptx.
