# Supplementary material for: Musculoskeletal Dimension and Brightness Reference Values in Lumbar Magnetic Resonance Imaging—A Radio-Anatomic Investigation in 80 Healthy Adult Individuals
Source: J Clin Med. 2024 Aug 1;13(15):4496. doi: 10.3390/jcm13154496 (PMC11313155; doi:10.3390/jcm13154496)
Supplement: Supplementary file 1 [file jcm-13-04496-s001.zip › jcm-3041216-supplementary.pptx]

## Slide 1
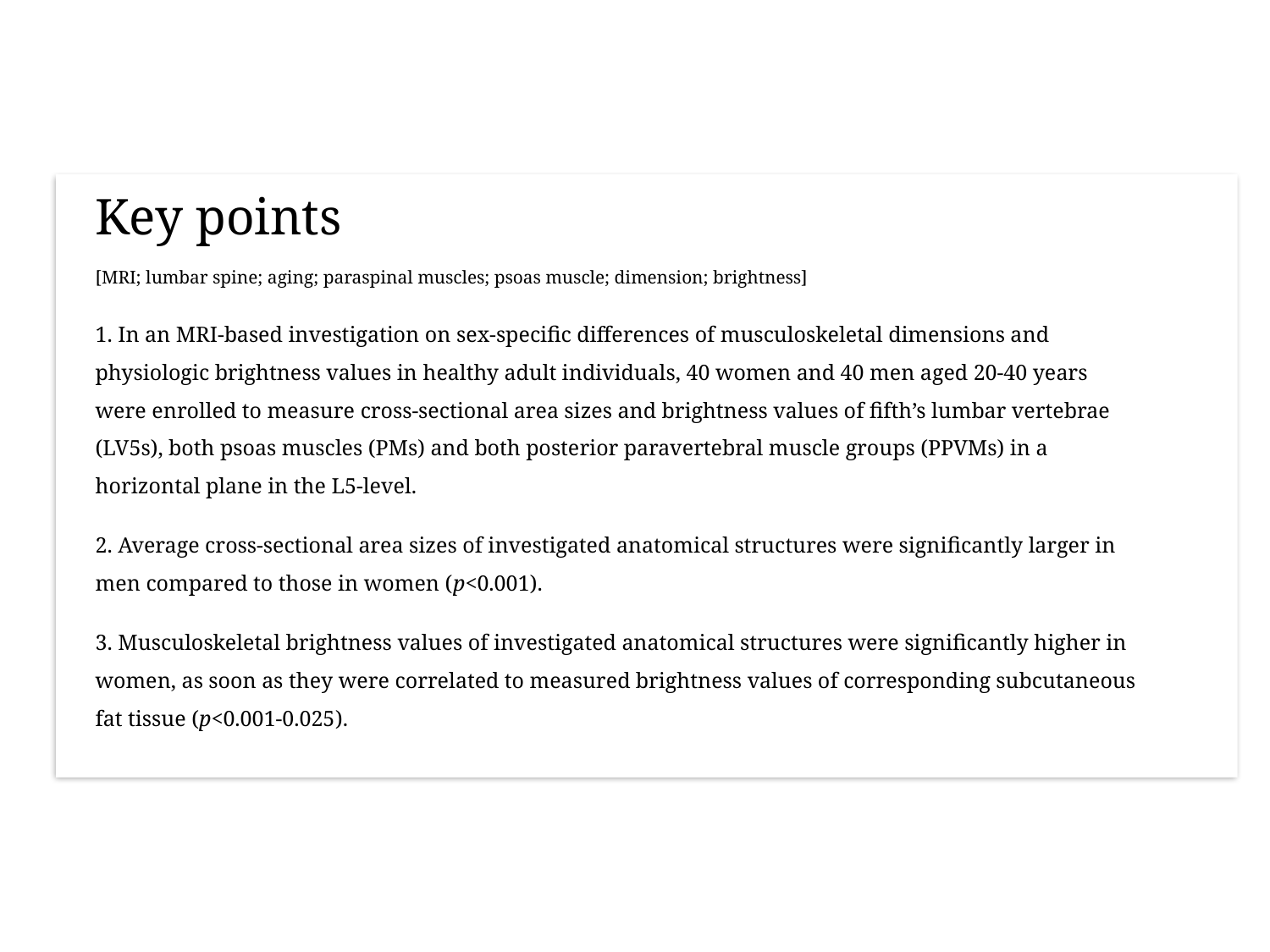

Key points
[MRI; lumbar spine; aging; paraspinal muscles; psoas muscle; dimension; brightness]
1. In an MRI-based investigation on sex-specific differences of musculoskeletal dimensions and physiologic brightness values in healthy adult individuals, 40 women and 40 men aged 20-40 years were enrolled to measure cross-sectional area sizes and brightness values of fifth’s lumbar vertebrae (LV5s), both psoas muscles (PMs) and both posterior paravertebral muscle groups (PPVMs) in a horizontal plane in the L5-level.
2. Average cross-sectional area sizes of investigated anatomical structures were significantly larger in men compared to those in women (p<0.001).
3. Musculoskeletal brightness values of investigated anatomical structures were significantly higher in women, as soon as they were correlated to measured brightness values of corresponding subcutaneous fat tissue (p<0.001-0.025).

## Slide 2
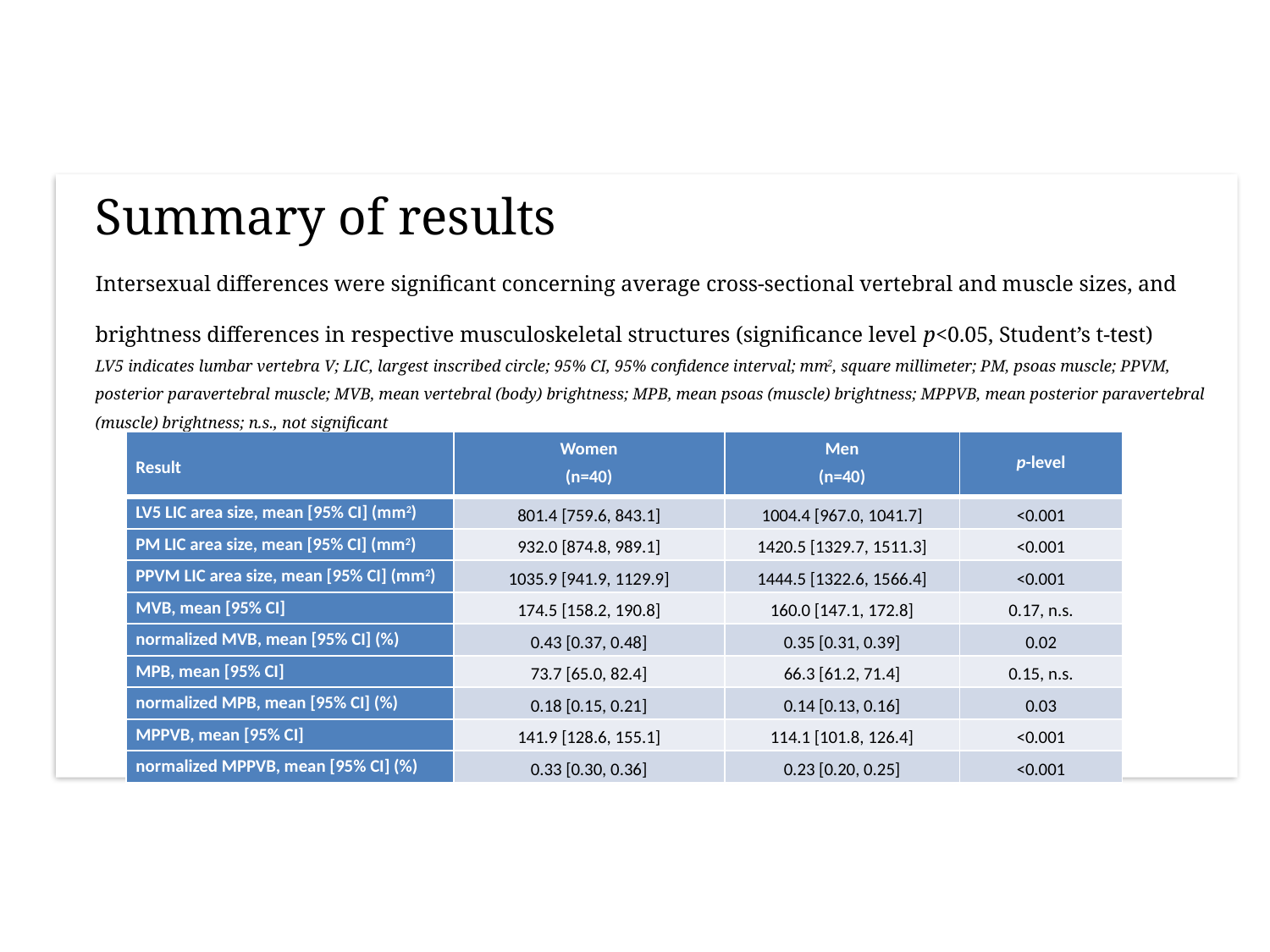

Summary of results
Intersexual differences were significant concerning average cross-sectional vertebral and muscle sizes, and brightness differences in respective musculoskeletal structures (significance level p<0.05, Student’s t-test)
LV5 indicates lumbar vertebra V; LIC, largest inscribed circle; 95% CI, 95% confidence interval; mm2, square millimeter; PM, psoas muscle; PPVM, posterior paravertebral muscle; MVB, mean vertebral (body) brightness; MPB, mean psoas (muscle) brightness; MPPVB, mean posterior paravertebral (muscle) brightness; n.s., not significant
| Result | Women (n=40) | Men (n=40) | p-level |
| --- | --- | --- | --- |
| LV5 LIC area size, mean [95% CI] (mm2) | 801.4 [759.6, 843.1] | 1004.4 [967.0, 1041.7] | <0.001 |
| PM LIC area size, mean [95% CI] (mm2) | 932.0 [874.8, 989.1] | 1420.5 [1329.7, 1511.3] | <0.001 |
| PPVM LIC area size, mean [95% CI] (mm2) | 1035.9 [941.9, 1129.9] | 1444.5 [1322.6, 1566.4] | <0.001 |
| MVB, mean [95% CI] | 174.5 [158.2, 190.8] | 160.0 [147.1, 172.8] | 0.17, n.s. |
| normalized MVB, mean [95% CI] (%) | 0.43 [0.37, 0.48] | 0.35 [0.31, 0.39] | 0.02 |
| MPB, mean [95% CI] | 73.7 [65.0, 82.4] | 66.3 [61.2, 71.4] | 0.15, n.s. |
| normalized MPB, mean [95% CI] (%) | 0.18 [0.15, 0.21] | 0.14 [0.13, 0.16] | 0.03 |
| MPPVB, mean [95% CI] | 141.9 [128.6, 155.1] | 114.1 [101.8, 126.4] | <0.001 |
| normalized MPPVB, mean [95% CI] (%) | 0.33 [0.30, 0.36] | 0.23 [0.20, 0.25] | <0.001 |

## Slide 3
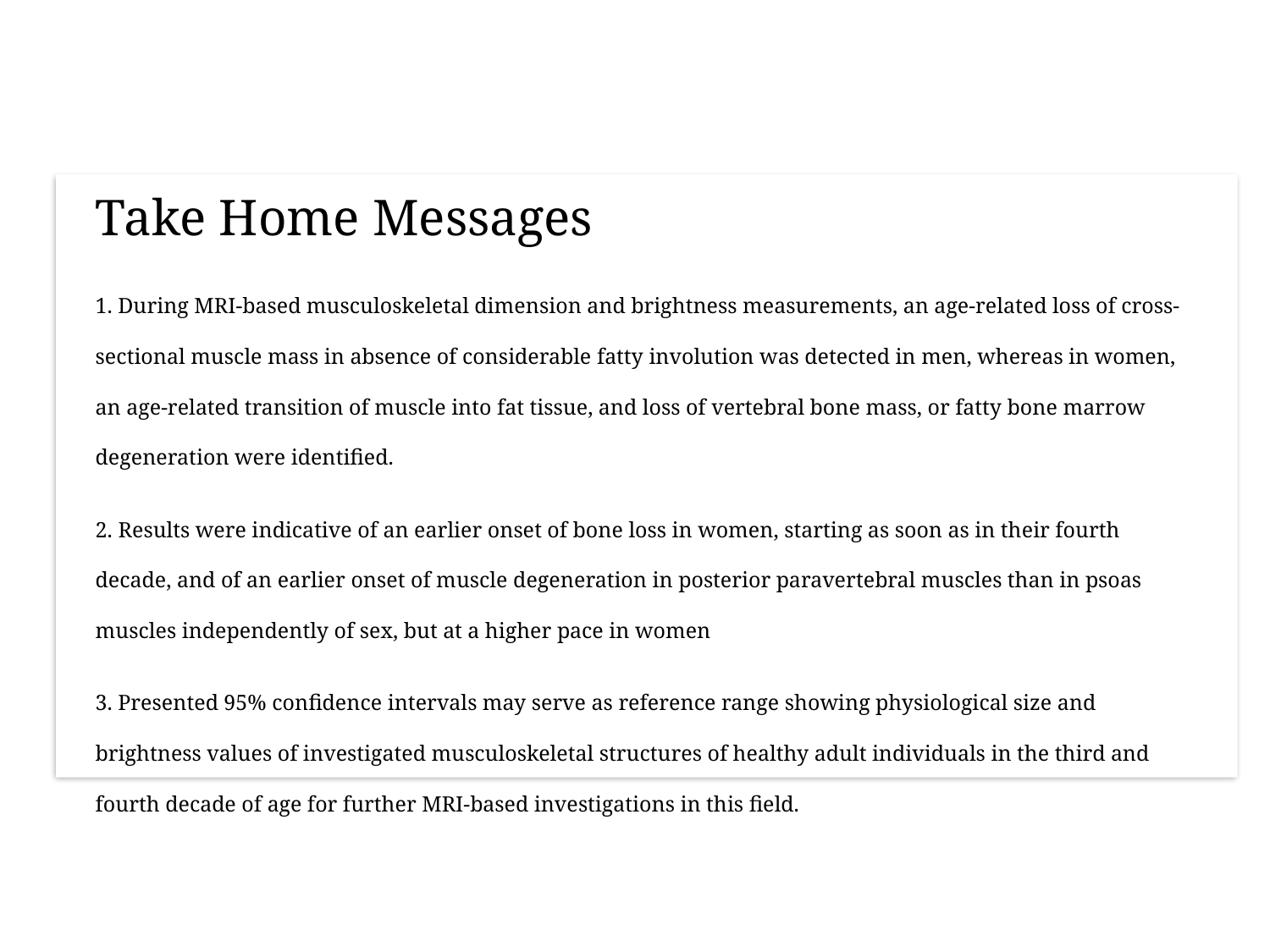

Take Home Messages
1. During MRI-based musculoskeletal dimension and brightness measurements, an age-related loss of cross-sectional muscle mass in absence of considerable fatty involution was detected in men, whereas in women, an age-related transition of muscle into fat tissue, and loss of vertebral bone mass, or fatty bone marrow degeneration were identified.
2. Results were indicative of an earlier onset of bone loss in women, starting as soon as in their fourth decade, and of an earlier onset of muscle degeneration in posterior paravertebral muscles than in psoas muscles independently of sex, but at a higher pace in women
3. Presented 95% confidence intervals may serve as reference range showing physiological size and brightness values of investigated musculoskeletal structures of healthy adult individuals in the third and fourth decade of age for further MRI-based investigations in this field.
